# Structure Characterization of Honey-Processed *Astragalus* Polysaccharides and Its Anti-Inflammatory Activity In Vitro

**DOI:** 10.3390/molecules23010168

**Published:** 2018-01-15

**Authors:** Jingzhu Liao, Chanyi Li, Jing Huang, Wuping Liu, Hongce Chen, Shuangye Liao, Hongyuan Chen, Wen Rui

**Affiliations:** 1 Centre Laboratory, Guangdong Pharmaceutical University, Guangzhou 510006, China; aprilljz@163.com (J.L.); lichanyi1994@163.com (C.L.); 15918743457@126.com (J.H.); 13424036867@163.cm (W.L.); liaoshy@sysucc.org.cn (S.L.); 2 Department of Pathogen Biology and Immunology, School of Basic Course, Guangdong Pharmaceutical University, Guangzhou 510006, China; shy@sysucc.org.cn (Hongc.C.); hychen@gdpu.edu.cn (Hongy.C.); 3 Key Laboratory of Digital Quality Evaluation of Chinese Materia Medica of State Administration of TCM, Guangzhou 510006, China; 4 Guangdong Engineering & Technology Research Center of Topical Precise Drug Delivery System, Guangdong Pharmaceutical University, Guangzhou 510006, China

**Keywords:** *Astragalus* polysaccharide, monosaccharide, UPLC/ESI-TOF-MS, anti-inflammatory activity

## Abstract

Honey-processed *Astragalus* is a dosage form of Radix *Astragalus* mixed with honey by a traditional Chinese medicine processing method which strengthens the tonic effect. *Astragalus* polysaccharide (APS), perform its immunomodulatory effects by relying on the tonic effect of Radix *Astragalus*, therefore, the improved pharmacological activity of honey-processed *Astragalus* polysaccharide (HAPS) might be due to structural changes during processing. The molecular weights of HAPS and APS were 1,695,788 Da, 2,047,756 Da, respectively, as determined by high performance gel filtration chromatography combined with evaporative light scattering detection (HPGFC-ELSD). The monosaccharide composition was determined by ultra-performance liquid chromatogram quadrupole time-of-flight mass spectrometry (UPLC/ESI-Q-TOF-MS) after pre-column derivatization with 1-phenyl-3-methyl-5-pyrazolone (PMP). The results showed that the essential components were mannose, glucose, xylose, arabinose, glucuronic acid and rhamnose, is molar ratios of 0.06:28.34:0.58:0.24:0.33:0.21 and 0.27:12.83:1.63:0.71:1.04:0.56, respectively. FT-IR and NMR analysis of HAPS results showed the presence of uronic acid and acetyl groups. The anti-inflammatory activities of HAPS were more effective than those of APS according to the NO contents and the expression of IFN-γ, IL-1β, IL-22 and TNF-α in lipopolysaccharide (LPS)-induced RAW264.7 cells. This findings suggest that the anti-inflammatory and bioactivity improvement might be associated with molecular structure changes, bearing on the potential immunomodulatory action.

## 1. Introduction

Radix *Astragalus*, the dry root of *Astragalus membranaceus* (Fisch.) Bge. var. mongholicus (Bge.) Hsiao or A. Membranaceus (Fisch.) Bge., has been used as a health-promoting herb in China for more than 2000 years [1,2]. *Astragalus* polysaccharide (APS), the main component of aqueous extracts of *Astragalus*, has various biological activities [3], including antitumor [4,5], antidiabetes [6,7], antiviral [8], anti-inflammatory [9], and in particular, immunomodulatory properties [10,11,12].

Honey-processed *Astragalus*, a dosage form of Radix *Astragalus* mixed with honey by a traditional Chinese medicine processing method, is used instead of Radix *Astragalus* to strengthen the efficacy in tonifying Qi [13], so the study of the structural differences between the honey-processed APS (HAPS) and APS, may reveal the material basis for the improved efficacy of HAPS in tonifying Qi. Moreover, our preliminary experiments confirmed that HAPS had a certain anti-inflammatory activity. The relationship between HAPS and this anti-inflammatory activity is unknown. Here, we investigate the molecular weight, monosaccharide composition and anti-inflammatory activity of HAPS to elucidate the relationships between its structure and its bioactivity and explain the distinction between HAPS and APS.

## 2. Results and Discussion

### 2.1. Determination of the Molecular Weight of HAPS by HPGFC-ELSD

The molecular weight of HAPS and APS were detected by HPGFC-ELSD, which offers high sensitivity and consistent analysis results. Dextrans standards (Mw: 12, 50, 150, 270 and 410 kDa) were used as they are water soluble and available in a wide range of molecular masses. These standards gave guidelines to estimate the size of HAPS. A standard curve was obtained (*y* = −0.27419*x* + 10.122, R^2^ = 0.99524), from which the differences between the HAPS and APS were determined. The results showed that the molecular weight of HAPS and APS were 1,695,788 Da and 2,047,756 Da, respectively.

### 2.2. Determining the Composition of Monosaccharide

#### 2.2.1. Monosaccharide Composition Determination

The monosaccharide composition of HAPS was determined by UPLC/ESI-Q-TOF-MS after pre-column derivatization with 1-phenyl-3-methyl-5-pyrazolone (PMP). The pre-column derivatization method attaches UV absorbing groups to the carbohydrates and changes the polarity, making it easy to separate the produced sugar derivatives [14].

The chromatographic separation of monosaccharides was performed on a UPLC system equipped with a Kinetex 1.7 μm column. The UPLC separation conditions were adjusted and tested for a mixture of six PMP-labelled monosaccharide standards (mannose, glucose, xylose, arabinose, glucuronic acid and rhamnose). During the mobile phase evaluation process, we found that the addition of a small amount of acid to the mobile phase accelerated the ionization of components, improving the peak shape and peak response intensity and minimizing peak tailing in the positive ion mode. A mobile phase containing acetonitrile also leads to improved peak shape. The best separation was obtained by gradient elution with 0.1% formic acid-10 mM ammonium acetate.

**Figure 1:**
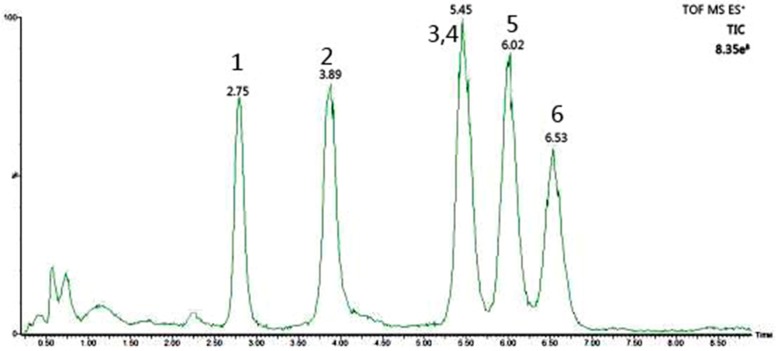
The total ion chromatograms of standard monosaccharides in positive ion mode (1: mannose, 2: rhamnose, 3: glucuronic, 4: glucose, 5: xylose, 6: arabinose).

**Figure 2:**
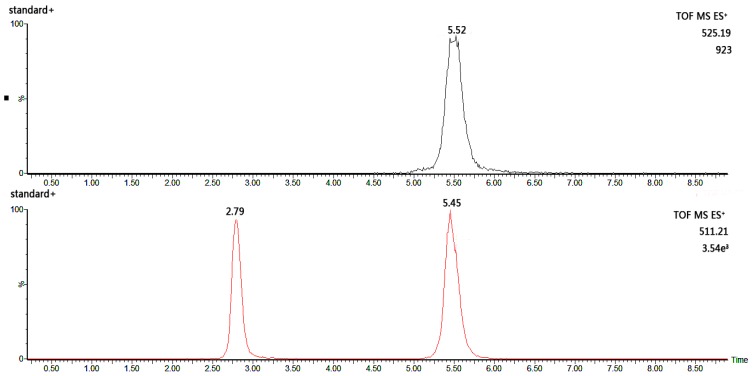
The extracted ion chromatograms of glucuronic acid and glucose.

Six PMP-labelled monosaccharidex were separated successfully: mannose (peak 1), rhamnose (peak 2), glucuronic acid (peak 3), glucose (peak 4), xylose (peak 5) and arabinose (peak 6). In this study, a good separation of the six monosaccharide derivatives was achieved within 7 min (Figure 1). The glucuronic acid and glucose peaks co-eluted from the column, and were better separated in the extracted ion chromatogram (EIC) based on their mass-to-charge ratio (Figure 2). The EICs of the six monosaccharide derivatives were used for determination of the monosaccharide contents.

#### 2.2.2. Method Validation for Monosaccharide Composition Testing

The RSD of relative retention time and relative peak area were used as the criteria for the validation of this method. Six consecutive injections of reference standards were used to assess the intra-day precision. As shown in Table 1 and Table 2, the RSDs of relative retention time and relative peak area for intra-day precision were less than 0.0078% and 0.1448%, respectively. The repeatability was assessed by detecting five different solutions prepared under the same conditions. The RSDs of relative retention time and relative peak area for repeatability was less than 0.0070% and 0.1270%, respectively. The calibration curves were constructed based on peak area versus concentrations of analyte standards.

Table 3 shows the summary of calibration curves, linear ranges. Good linearities were found in the ranges of 0.1–10 nmol. These findings indicated that the established method was reliable and useful for the analysis of HAPS and APS.

#### 2.2.3. Monosaccharide Composition of HAPS

The established UPLC/ESI-Q-TOF-MS method was then applied to analyze the constituents of HAPS and APS. The raw data files were processed with the Masslynx 4.1 software, which provided the elemental compositions and mass errors. The elemental compositions were accepted only when the deviation was within 5 mDa.

**Figure 3:**
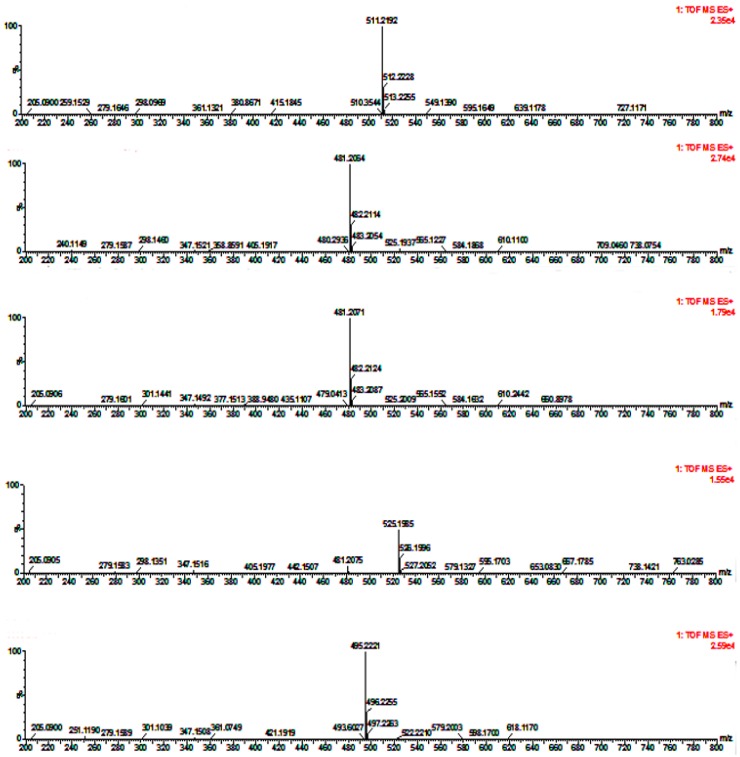
Mass spectra of reference monosaccharides (from the **top** to the **bottom** they are mannose, glucose, xylose, arabinose, glucuronic acid and rhamnose, respectively).

We identified the monosaccharides of pure HAPS and APS by comparing their retention times and mass spectra data with those of the standards (Figure 3). The results showed that both the HAPS and the APS have the same six monosaccharides: Mannose, glucose, xylose, arabinose, glucuronic acid and rhamnose but their relative contents are different

**Figure 4:**
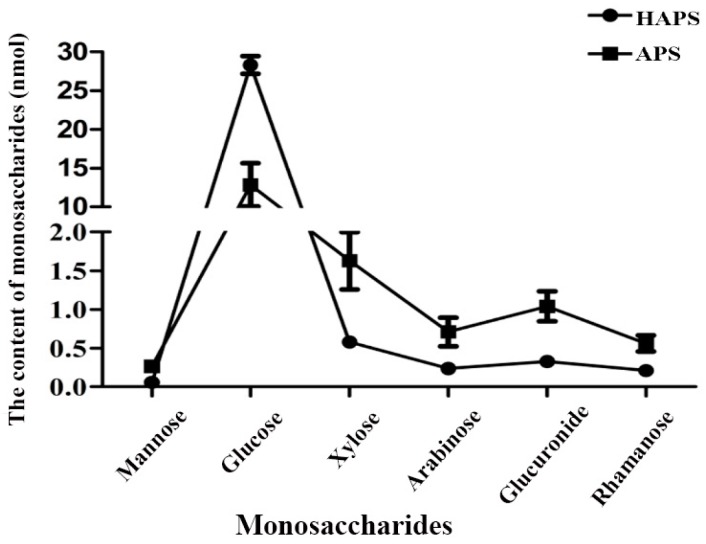
The monosaccharide proportions in HAPS and APS.

As shown in Figure 4, glucose was the main monosaccharide in both HAPS and APS. The amount of glucose in HAPS was almost twice as much as that in APS, while the other monosaccharides of HAPS was less than those of APS. The results showed that the composition ratio of monosaccharides (nmol) in HAPS was mannose:glucose:xylose:arabinose:glucuronic acid:rhamnose = 0.06:28.34:0.58:0.24:0.33:0.21, while the composition ratio of monosaccharides (nmol) in APS was 0.27:12.83:1.63:0.71:1.04:0.56. The larger ratio of glucose in HAPS might be due to the honey-processed procedure which made the polysaccharides bind to glucose released from honey. In the honey-processing procedure, the high temperature might lead to the degradation of the polysaccharide [15,16], so the other monosaccharides might decrease accordingly.

### 2.3. FT-IR and NMR Analysis

**Figure 5:**
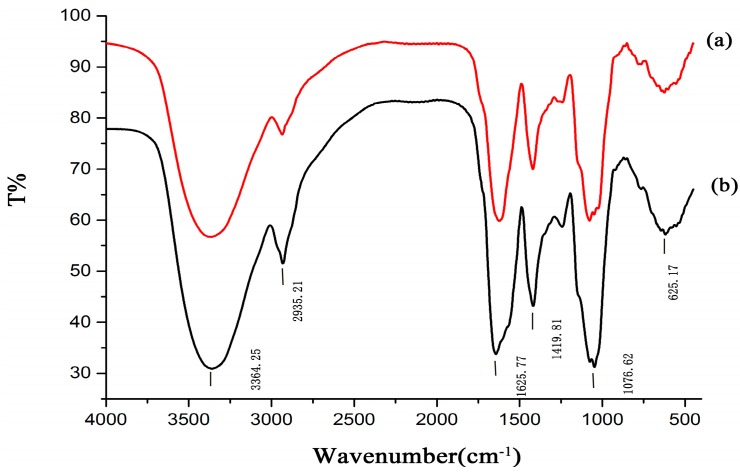
FT-IR spectra of HAPS (**b**) and APS (**a**), the spectral measurement within 4000–450 cm^−1^.

The infrared spectra of HAPS and APS are shown in Figure 5. The figure indicates that the polysaccharide spectra had a strong and wide O-H stretching vibration band at 3364.25 cm^−1^ which reflects the intense -inter and -intra molecular interactions of the polysaccharide. The weak absorption at 2935.31 cm^−1^ correspond to the C-H stretching vibration. The strong absorption peak at 1625.77 cm^−1^ corresponding to ester carbonyl and carboxyl groups, which indicates the possible presence of uronic acid in the polysaccharide.

Compared with APS, the peak pattern and intensity were changed slightly, including the peaks at 1625.77 cm^−1^ and 1076.62 cm^−1^, which represent uronic acid and C-O stretching vibrations, respectively.

**Figure 6:**
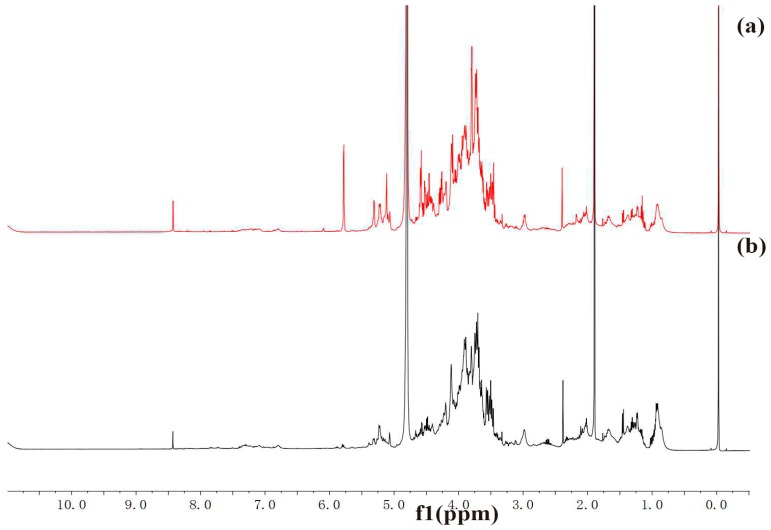
^1^H-NMR spectra of HAPS (**b**) and APS (**a**).

The ^1^H-NMR spectra of HAPS and APS are shown in Figure 6. The anomeric proton signals at δ 3.5 ppm correspond to methoxy protons, and signals at δ 2.0 ppm correspond to acetyl group protons. Compared with APS, the acetyl proton signal of HAPS was stronger. Based on monosaccharide analysis, the anomeric proton signals at δ 5.12 ppm correspond to the H-1 of β-arabinose, the anomeric proton signals at δ 4.51 ppm correspond to the H-1 of β-glucose, the anomeric proton signals at δ 5.09 ppm correspond to the H-1 protons of α-glucose or α-xylose. However, the spectrum was overlapped entirely, so no further details could be extracted.

The FT-IR and ^1^H NMR spectrum information indicated that the difference between HAPS and APS is mostly the intensity of the uronic acid and acetyl groups. Moreover, the comparison information between the spectra and the monosaccharide composition provides mutual verification of the results.

### 2.4. Anti-Inflammatory Activity of HAPS In Vitro

#### 2.4.1. Effects of the HAPS on Cell Viability and Inhibition of NO Expression in LPS-Stimulated RAW264.7 Cells

The cytotoxic activities of the HAPS were determined by an MTT assay. The concentrations which ranged from 10 to 300 μg mL^−1^ of the HAPS did not influence the cell viability, and the cell viability of 100 μg mL^−1^ of the HAPS was almost to 100%.

We measured the LPS-induced production of NO as indicator to assess the anti-inflammatory effects of the HAPS. The amount of NO in cell supernatants after 24 h of treatment with LPS in the presence or absence of various doses of the HAPS (10, 50, 100, 300 μg mL^−1^) were determined by ELISA. Generally, as shown in Table 4, the LPS induced production of NO were considerably restrained by the HAPS in a dose-dependent manner and HAPS has stronger inhibition g than APS does. Indeed, even the lowest dose of the HAPS and APS (10 μg mL^−1^) inhibits the production of NO.

#### 2.4.2. Effects of HAPS on IFN-γ, IL-1β, IL-22 and TNF-α Expression in LPS-Stimulated RAW264.7 Cells

**Figure 7:**
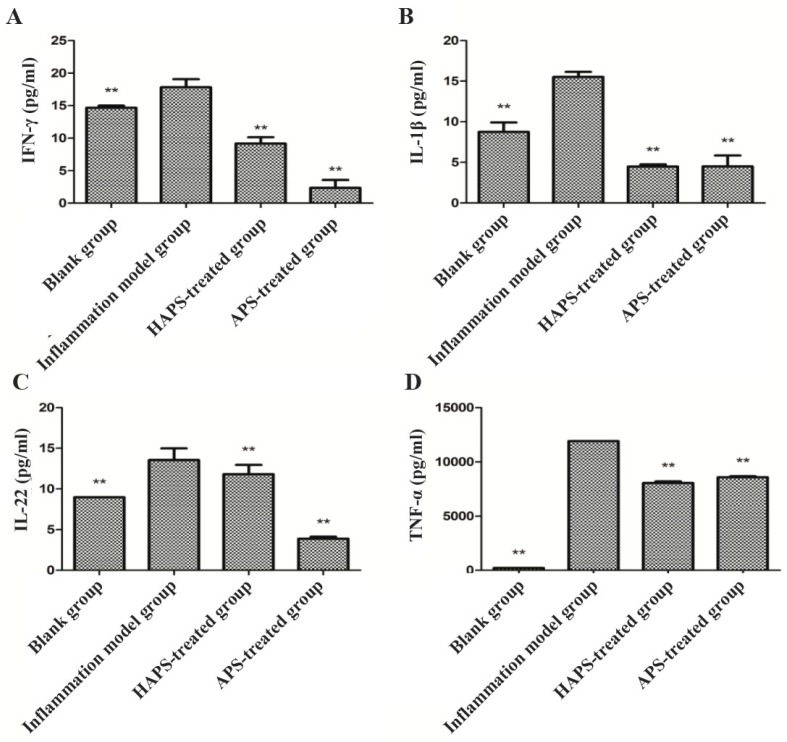
Effects of HAPS and APS on the expression of inflammatory cytokines IFN-γ (**A**); IL-1β (**B**); IL-22 (**C**) and TNF-α (**D**) in LPS-stimulated RAW264.7 cells. * Indicates the statistical significance of the difference between inflammation model group and blank group, honey-processed-treated group, APS-treated group and Ibuprofen-treated group (*n* = 6, ** *p* < 0.01 vs. inflammation model group).

RAW264.7 cells are macrophages widely used in in vitro models for inflammatory studies [17]. Once RAW264.7 cells are activated by LPS, they elicit proinflammatory cytokines, including NO, IFN-γ, IL-1β, IL-22 and TNF-α mediated by upstream inflammation-associated genes. All of the above are inflammatory cytokines which could bind to cell surface receptors and elicit signals in cells, activating afterwards some transcription factors like NF-κB [17], JAK/STAT [18], causing all kinds of interaction between transcription factors, activating the expression of inflammatory cytokines, and triggering various biological effects. The protein expressions of IFN-γ, IL-1β, IL-22 and TNF-α are appropriate indexes to evaluate the degree of inflammatory activity. The results showed that LPS strongly increased the expression of IFN-γ, IL-1β, IL-22 and TNF-α compared to those of the blank group (Figure 7). However, HAPS and APS treatment decreased the expression of these four pro-inflammatory mediators when co-incubated with LPS treated cells. Compared with APS, the anti-inflammatory effects of HAPS are better. This might be due to the molecular weight variation and different monosaccharide composition ratio.

## 3. Experimental

### 3.1. Materials and Reagents

HPLC-grade acetonitrile, methanol, and isopropanol were purchased from Merck (Darmstadt, Germany). Formic acid of HPLC grade was from CNW Technologies GmbH (Düsseldorf, Germany). Trifluoroacetic acid and 1-phenyl-3-methyl-5-pyrazolone were obtained from Sigma (Cream Ridge, NJ, USA). All water was purified by a water purification system (18.2 MΩ, Sartorius, Goettingen, Germany).

Standard dextrans (Mw: 12, 50, 150, 270 and 410 kDa) were purchased from Sigma (Marin-Epagnier, Switzerland). Glucuronic acid was from Tokyo Chemical (Tokyo, Japan), while Glucose, mannose, xylose, rhamnose and arabinose were from Qiyun (Guangzhou, China). Dulbecco’s modified Eagle’s medium with high glucose and Fetal Bovine Serum were purchased from Gibco (Langley, OK, USA), as was phosphate buffered saline. Antibiotic (penicillin/ streptomycin) solution was obtained from Solarbio (Beijing, China). 3-(4,5-Dimethyl-thiazol-2-yl)-2,5-diphenyltetrazolium bromide (MTT), LPS, and dimethyl sufoxide (DMSO) were purchased from Sigma (USA). Mouse Inducible Nitric Oxide Synthase (iNOS) ELISA Kit, and Mouse IFN-γ, IL-1β, IL-22 and TNF-α ELISA kits were purchased from Multisciences (Hangzhou, China). *Astragalus membranaceus* (Fisch.) Bge. Var. mongholicus (Bge.) Hsiao was purchased from Cai Zhilin (Guangzhou, China) and identified by Professor JizhuLiu in Guangdong Pharmaceutical University. All the samples were preserved in our laboratory.

### 3.2. Sample Preparation

The honey-processed *Astragalus* and radix *Astragalus* were crushed into powders, respectively. The polysaccharide was isolated from *Astragalus* by hot-water extraction and ethanol precipitation [19]. The dried powder was first immersed in 70% ethanol at 80 °C for 1 h to remove the pigments and small organic compounds, then extracted with distilled water. The water extracts were concentrated under reduced pressure. Then the collected supernatants were precipitated with 70% (*v*/*v*) ethanol at 4 °C overnight after concentration. Dissociative protein was removed by Sevage method [20] and then purified by gel chromatography.

### 3.3. Determination of the Molecular Weight of the Polysaccharides 

The chromatographic separation was performed using a Polysep-GFC-P4000 column (300 × 7.8 mm, Phenomenex, Torrance, CA, USA) equipped with a Polysep-GFC-P pre-Column (35 × 7.8 mm) at a column temperature of 30 °C and liquid flow rate of 0.3 mL/min. The mobile phase was water, the injection volume was 10 μL with a partial loop and an overfilled needle. The adopted ELSD conditions for the analysis of all polysaccharides were: 100 °C drift tube temperature, 60 °C nebulizer temperature, 30 psi gas pressure, 10 pps date rate and 10 as the gain factor. Standard dextrans (Mw: 12, 50, 150, 270 and 410 kDa) with concentration 10 mg/mL were applied as the standard solution. A standard curve was obtained for the molecular weight calculation [21]. Under the experiment conditions, the diversity data was available for the HAPS and APS by running them on the Waters Empower 3.0 software.

### 3.4. Hydrolysis and PMP Derivatization

The polysaccharide sample (10 mg) was added with 2 mL 4 M TFA in a sealed tube (10 mL) and kept at 100 °C oil bath for 8 h. Then it was prepared for derivatization after evaporating with methanol to dry the residual TFA, and freeze drying. The procedure employed for the derivatization of monosaccharide was carried out following the methods of Daotian et al. [22]. The hydrolysed polysaccharide or monosaccharides were dissolved in 5 mL ammonia. Then, the 100 μL solution was transferred into a clean tube, and 0.5 M methanol solution (100 μL) of PMP was added and mixed. The mixture cooled to ambient temperature after reaction for 30 min at 70 °C, then freeze-dried. These derivatization processes should be repeated twice [23]. One mL of methanol was added to dissolve the freeze-dried samples. Then, the supernatant was collected for UPLC/Q-TOF-MS analysis directly after centrifugation at 10,000 rpm for 5 min.

### 3.5. UPLC/Q-TOF-MS Analysis of the Composition of Monosaccharide

The UPLC separation was performed at 25 °C using a Kinetex 1.7 μm EVO C18 column (2.1 mm × 50 mm, 1.7 μm, Phenomenex). The mobile phase consisted of acetonitrile (A) and water with 0.1% (*v*/*v*) formic acid-10 mM ammonium acetate (B) with a gradient elution: 0–10 min, 85–85% A; 10–14 min, 85–30% A; 14–15 min, 30–85%; 15–17 min, 85–85%. The injection volume was 2 μL at a flow rate of 0.4 mL min^−1^.

Mass detection was performed in the full scan mode at the *m*/*z* range from 100 to 600 with positive ionization mode. The nebulizer gas (N_2_) and the desolvation gas (N_2_) were at flow rates of 50 L h^−1^ and 500 L h^−1^, respectively. The source and desolvation temperatures were set at 100 °C and 300 °C, respectively. The capillary voltages were 3.0 kV, and the cone voltages were 30 V. Leucine enkephalin was used as the lock mass ([M + H]^+^ at *m*/*z* 556.2771) to ensure mass accuracy and reproducibility at a flow rate of 0.02 mL min^−1^. The lock spray frequency was set at 10 s. All data acquisition and processing were conducted with the Masslynx 4.1 software incorporated in the instrument.

### 3.6. Method Validation for Monosaccharide Composition Testing

Our methods were validated after optimizing the UPLC/Q-TOF-MS conditions. A series of standard solutions evaluated the linearity of each analyte. The calibration curves were constructed based on the peak area versus concentrations values of analyte standards. The standard mixture solution was analyzed six times a day under the optimum conditions for intra-day variation to estimate precision and accuracy. Five different solutions, prepared as described in Section 2.4 from the same sample, were detected to check the repeatability.

### 3.7. FT-IR and NMR Analysis

IR spectra of HAPS and APS were recorded using the KBr-disk method with a Fourier transform infrared (FT-IR) spectrometer (Spectrum 100, PerkinElmer Co., Ltd., Waltham, MA, USA). The spectral measurement within 4000–450 cm^−1^. HAPS and APS were dissolved in D_2_O at a concentration of 60 mg/mL for recording the corresponding ^1^H-NMR spectra on a 500 MHz Bruker spectrometer (Bruker, Biospin, Switzerland).

### 3.8. Cell Viability Assay & Measurement of NO Release

The cytotoxicity of the HAPS and APS in RAW264.7 cells were evaluated through the MTT assay. Dependent on the activation degree of cells, the signal was read in a microplate spectrophotometer, which was usually applied to measure cytotoxicity through optical density values [24]. Different levels of NO have been shown to be associated with inflammatory diseases [25]. As NO is a gaseous free radical with a short half-life, the levels of the more stable metabolites, nitrite and nitrate were measured as indicators of the released NO in LPS-induced RAW264.7 cells to investigate the anti-inflammatory effect of the HAPS and APS. Log phase RAW264.7 cells were placed in 96-well plates. Subsequently, these cells were treated with LPS, the HAPS and APS (10, 50, 100, 300 μg mL^−1^) or ibuprofen (10, 50, 100, 300 μg mL^−1^) for 24 h. Afterwards, the release of NO in supernatant was determined by a Total Nitric Oxide and Nitrate/Nitrite Assay kit under the manufacturer’s directions. The absorbance was measured by microplate spectrophotometer at 540 nm.

### 3.9. ELISA for the Detection of IFN-γ, IL-1β, IL-22 and TNF-α Cytokines Expression

Combined with the results from Section 3.8, concentration of HAPS and APS were chosen as 100 μg mL^−1^. Log phase RAW264.7 cells were placed in 24-well plates. Subsequently, the cells were treated with 100 μg mL^−1^ HAPS and APS for 24 h. Afterwards, the release of IFN-γ, IL-1β, IL-22 and TNF-α in supernatant were determined by ELISA kits following the manufacturer’s directions. The absorbance was measured by a microplate spectrophotometer with excitation wave length at 620 nm.

## 4. Conclusions

The UPLC-Q-TOF-MS method after pre-column derivatization with PMP established in this study shows higher stability and repeatability compared to the traditional method of analysis of monosaccharide composition. Our method could also be used for quality control of HAPS and provide an optional method for the quality control of *Astragalus* in traditional Chinese herbs. The results showed that the molecular weights of HAPS declines and the monosaccharide composition ratio changed. In the honey-processing procedure, carbohydrates might be degraded, while the intermolecular hydrogen bonding interaction might be enhanced, which causes the variation of molecular weight and the monosaccharide composition of HAPS. Furthermore, the better anti-inflammatory activity of HAPS might be closely related to its structure variation. These studies lay the foundation for the next phrase of research. Our experimental results provide a preliminary experimental basis for the study of structure-activity relationships of HAPS, and further pharmacological effects should be verified by animal models in the next experiments.

## Figures and Tables

**Table 1:** The statistical results of the intra-day precision of the monosaccharides (*n* = 6).

Monosaccharides	Relative Retention Time (RSD, %)	Relative Peak Area (RSD, %)
Mannose	0.00	0.09
Glucose	0.00	0.11
Xylose	0.00	0.12
Arabinose	0.00	0.14
Glucuronic acid	0.00	0.12
Rhamnose	0.01	0.10

**Table 2:** Statistical results of the reference monosaccharides (*n* = 5).

Monosaccharides	Relative Retention Time (RSD, %)	Relative Peak Area (RSD,%)
Mannose	0.00	0.09
Glucose	0.00	0.12
Xylose	0.01	0.06
Arabinose	0.01	0.12
Glucuronic acid	0.01	0.11
Rhamnose	0.00	0.13

**Table 3:** The calibration ranges for the reference monosaccharides.

Monosaccharide	Standard Curve (*n* = 6)	R^2^ (*n* = 6)	Linear Range (nmol)
Mannose	*y* = 4.2699*x* − 2.8281	R^2^ = 0.99132	0.1~10
Glucose	*y* = 3.2251*x* − 7.9961	R^2^ = 0.99781	0.1~10
Xylose	*y* = 7.2628*x* − 17.148	R^2^ = 0.99706	0.1~10
Arabinose	*y* = 5.0057*x* − 13.844	R^2^ = 0.99844	0.1~10
Glucuronic acid	*y* = 2.2763*x* − 5.5898	R^2^ = 0.99492	0.1~10
Rhamnose	*y* = 6.4915*x* − 22.234	R^2^ = 0.99614	0.1~10

**Table 4:** Results of NO release after treatment of different concentration HAPS and APS.

Treatment Groups (μg mL^−1^)		NO Release Rate/μM
Blank group		0.84 ± 0.50 **
Inflammation model group		5.28 ± 0.37
HAPS	10	2.80 ± 0.43 **
	50	2.75 ± 0.66 **
	100	2.44 ± 1.04 **
	300	2.06 ± 1.13 **
APS	10	3.92 ± 0.98 **
	50	2.97 ± 0.67 **
	100	2.81 ± 0.47 **
	300	2.42 ± 0.23 **
Ibuprofen	10	5.19 ± 0.001
	50	4.87 ± 0.004
	100	4.27 ± 0.003 *
	300	2.93 ± 0.002 **

Data are presented as mean ± S.D. from experiments repeated 6 times. * Indicates the statistical significance of the difference among inflammation model group and blank group, HAPS-treated group, APS-treated group and ibuprofen-treated group (*n* = 6, * *p* < 0.05, ** *p* < 0.01 vs. inflammation model group).

## References

[1] Pharmacopoeia Commission of PRC (2015). Pharmacopoeia of the People’s Republic of China.

[2] Ma X.Q., Shi Q., Duan J.A., Dong T.T., Tsim K.W. (2002). Chemical Analysis of Radix *Astragalus* (Huangqi) in China: A Comparison with Its Adulterants and Seasonal Variations. Food Chem..

[3] Jin M., Zhao K., Huang Q., Shang P. (2014). Structural features and biological activities of the polysaccharides from *Astragalus membranaceus*. Int. J. Biol. Macromol..

[4] Yang B., Xiao B., Sun T. (2013). Antitumor and immunomodulatory activity of *Astragalus membranaceus* polysaccharides in H22 tumor-bearing mice. Int. J. Biol. Macromol..

[5] Sun S., Zheng K., Zhao H., Lu C., Liu B., Yu C., Zhang G., Bian Z., Lu A., He X. (2014). Regulatory Effect of *Astragalus* Polysaccharides on Intestinal Intraepithelial γδT Cells of Tumor Bearing Mice. Molecules.

[6] Zhao M., Zhang Z.F., Ding Y., Wang J.B., Li Y. (2012). Astragalus Polysaccharide Improves Palmitate-Induced Insulin Resistance by Inhibiting PTP1B and NF-κB in C2C12 Myotubes. Molecules.

[7] Dun C., Liu J., Qiu F., Wu X., Wang Y., Zhao Y., Gu P. (2016). Effects of *Astragalus* polysaccharides on memory impairment in a diabetic rat model. Neuropsychiatr. Dis. Treat..

[8] Jiang J., Wu C., Gao H., Song J., Li H. (2010). Effect of *Astragalus* polysaccharides on immunologic function of erythrocyte in chickens infected with infectious bursa disease virus. Vaccine.

[9] Wei W., Xiao H.T., Bao W.R., Ma D.L., Leung C.H., Han X.Q., Ko C.H., Lau C.B.S., Chun-Kwok W.O.N.G., Fung K.P. (2016). TLR-4 may mediate signaling pathways of Astragalus polysaccharide RAP induced cytokine expression of RAW 264.7 cells. J. Ethnopharmacol..

[10] Zhou L., Liu Z., Wang Z., Yu S., Long T., Zhou X., Bao Y. (2017). *Astragalus* polysaccharides exerts immunomodulatory effects via TLR4-mediated MyD88-dependent signaling pathway in vitro and in vivo. Sci. Rep..

[11] Gao Y.J., Zhu F., Qian J.M., Dai J.Y. (2016). Therapeutic and Immunoregulatory Effect of GATA-Binding Protein-3/T-Box Expressed in T-Cells Ratio of *Astragalus* Polysaccharides on 2,4,6-Trinitrobenzene Sulfonic Acid-Induced Colitis in Rats. Chin. J. Integr. Med..

[12] Wang Z., Liu Z., Zhou L., Long T., Zhou X., Bao Y. (2017). Immunomodulatory effect of APS and PSP is mediated by Ca2+-cAMP and TLR4/NF-kB signaling pathway in macrophage. Int. J. Biol. Macromol..

[13] Xiao M., Chen H., Shi Z., Feng Y., Rui W. (2014). Rapid and reliable method for analysis of raw and honey-processed *Astragalus* by UPLC/ESI-Q-TOF-MS using HSS T3 columns. Anal. Methods.

[14] Honda S. (1996). Posteolumn derivatization for chromatographic analysis of carbohydrates. J. Chromatogr. A.

[15] Cai J.F., Dai Y.T., Xiao Y.Q., Zhao R., Zhang L.W. (2016). Systemic Evaluation of Effect of Honey-processing on Therapeutical Basis of *Astragalus* Radix. Chin. J. Exp. Tradit. Med. Formul..

[16] Ni D.J., Chen Y.Q., Xie B.J., Zhang Y., Zhou J.R. (2004). Spectrum, Morphological and Thermal Characteristics of OTPS 2-1 in Polysaccharides from Oolong Tea. Chem. Res. Chin. Univ..

[17] Lv J., Zhang Y., Tian Z., Liu F., Shi Y., Liu Y., Xia P. (2017). *Astragalus* polysaccharides protect agansit dextran sulfate sodium-induced colitis by inhibiting NF-κB, activation. Int. J. Biol. Macromol..

[18] Villarino A.V., Kanno Y., Ferdinand J.R., O’Shea J.J. (2015). Mechanisms of Jak/STAT signaling in immunity and disease. J. Immunol..

[19] Yin J.Y., Chan B.C.L., Yu H., Lau I.Y.K., Han X.Q., Cheng S.W., Wong C.K., Lau C.B.S., Xie M.Y., Fung K.P. (2012). Separation, structure characterization, conformation and immunomodulating effect of a hyperbranched heteroglycan from Radix Astragali. Carbohydr. Polym..

[20] Pu X., Ma X., Liu L., Ren J., Li H., Li X., Yu S., Zhang W., Fan W. (2016). Structural characterization and antioxidant activity in vitro of polysaccharides from angelica and astragalus. Carbohydr. Polym..

[21] Kärkkäinen J., Lappalainen K., Joensuu P., Lajunen M. (2011). HPLC-ELSD analysis of six starch species heat-dispersed in [BMIM]Cl ionic liquid. Carbohydr. Polym..

[22] Fu D.T., Oneill R.A. (1995). Monosaccharide composition analysis of oligosaccharides and glycoproteins by high-performance liquid chromatography. Anal. Biochem..

[23] Lin X., Wang Z., Huang L.J., Bai Q., Jia J.F. (2006). An improved PMP derivatization method for analyzing monosaccharide composition. Chem. J. Chin. Univ. Chin. Ed..

[24] Mosmann T. (1983). Rapid colorimetric assay for cellular growth and survival: Application to proliferation and cytotoxicity assays. J. Immunol. Methods.

[25] Miles A.M., Wink D.A., Cook J.C., Grisham M.B. (1996). Determination of nitric oxide using fluorescence spectroscopy. Methods Enzymol..

